# Lung Ultrasound Findings and Endothelial Perturbation in a COVID-19 Low-Intensity Care Unit

**DOI:** 10.3390/jcm11185425

**Published:** 2022-09-15

**Authors:** Roberta Gualtierotti, Francesco Tafuri, Raffaella Rossio, Matteo Rota, Paolo Bucciarelli, Barbara Ferrari, Andrea Giachi, Chiara Suffritti, Massimo Cugno, Flora Peyvandi, on behalf of the PRINCIPLUS Study Group

**Affiliations:** 1 Dipartimento di Fisiopatologia Medico-Chirurgica e dei Trapianti, Università degli Studi di Milano, 20122 Milan, Italy; 2 Fondazione IRCCS Ca’ Granda, Ospedale Maggiore Policlinico di Milano, UOC Medicina Interna—Emostasi e Trombosi, 20122 Milan, Italy; 3 Dipartimento di Medicina Molecolare e Traslazionale, Università degli Studi di Brescia, 25123 Brescia, Italy

**Keywords:** COVID-19, lung ultrasound, endothelial perturbation, low-intensity care, thrombomodulin, compression ultrasound

## Abstract

Hypercoagulability and endothelial dysfunction related to inflammation have been clearly demonstrated in COVID-19. However, their influence on thromboembolism, lung alterations and mortality in low-intensity-care patients with COVID-19 is not completely clarified. Our aims were to evaluate the prevalence of deep vein thrombosis (DVT) with compressive ultrasound (CUS); to describe lung ultrasound (LUS) features; and to study coagulation, inflammatory and endothelial perturbation biomarkers in COVID-19 patients at low-intensity care unit admission. The predictive value of these biomarkers on mortality, need for oxygen support and duration of hospitalization was also evaluated. Of the 65 patients included, 8 were non-survivors. CUS was negative for DVT in all patients. LUS Soldati and Vetrugno scores were strongly correlated (rho = 0.95) with each other, and both significantly differed in patients who needed oxygen therapy vs. those who did not (Soldati *p* = 0.017; Vetrugno *p* = 0.023), with coalescent B lines as the most prevalent pattern in patients with a worse prognosis. Mean (SD) levels of thrombomodulin and VCAM-1 were higher in non-survivors than in survivors (7283.9 pg/mL (3961.9 pg/mL) vs. 4800.7 pg/mL (1771.0 pg/mL), *p* = 0.004 and 2299 ng/mL (730.35 ng/mL) vs. 1451 ng/mL (456.2 ng/mL), *p* < 0.001, respectively). Finally, in a multivariate analysis model adjusted for age, sex and Charlson score, VCAM-1 level increase was independently associated with death [OR 1.31 (1.06, 1.81; *p* = 0.036)]. In conclusion, in a cohort of mild COVID-19 patients, we found no DVT events despite the highly abnormal inflammatory, endothelial and coagulation parameters. The presence of lung alterations at admission could not predict outcome. The endothelial perturbation biomarker VCAM-1 emerged as a promising prognostic tool for mortality in COVID-19.

## 1. Introduction

The clinical presentation of severe acute respiratory syndrome—coronavirus 2 (SARS-CoV-2) infection varies from asymptomatic cases to interstitial pneumonia with respiratory failure and severe disease and has been defined as coronavirus disease-19 (COVID-19) [1]. The infection is characterized by an exaggerated inflammatory response to the virus, described as a cytokine storm, with the consequent and concomitant activation of coagulation. In the first wave of the pandemic, these events combined with direct lung injury were considered to contribute not only to the development of hypoxia and acute respiratory distress syndrome (ARDS) [2] but also to coagulopathy with high incidence of thrombotic events such as deep venous thrombosis (DVT) and pulmonary embolism (PE) [3]. However, the role of DVT in contributing to PE in low-intensity-care COVID-19 patients is still debated, particularly in the subsequent disease waves, and the prevalence of DVT on hospital admission in these patients is not clear. The links between viral infection, inflammation and thrombosis could be represented by endothelial cell perturbation induced by cytokines. It has been demonstrated that the degree of endotheliopathy is correlated with severity and death in COVID-19 patients admitted in intensive care units (ICU) [4,5].

Chest computed tomography (CT) is the most sensitive imaging technique for assessing SARS-CoV-2 pneumonia [3] and diagnosing PE. During the pandemic period, lung ultrasound (LUS) has been considered a valuable tool for the diagnosis and follow-up of COVID-19 pneumonia in the emergency department, for bedside monitoring in critically ill patients and for defining the degree of pulmonary involvement [6,7]. The use of LUS has been standardized with the introduction of specific scores based on the different ultrasonographic patterns that have been described in SARS-CoV-2 infection [8,9]. The implementation of LUS and compressive ultrasound (CUS) in the clinical approach to COVID-19 patients has improved the clinical monitoring and differential diagnosis of respiratory failure, in particular in intensive care unit patients [10].

Different coagulation, inflammation and endothelial perturbation biomarkers have been studied in particular in severe COVID-19 patients admitted to intensive care. Notably, an incremental gradient in biomarker levels has been reported in COVID-19 patients with increasing disease severity [3,11].

With this as background, the primary endpoint of this study was to evaluate the prevalence of DVT on hospital admission via two-point femoral popliteal CUS in a low-intensity-care hospital ward. The secondary endpoint of the study was to describe LUS features in the same population of COVID-19 patients using two different LUS scores to predict mortality, time to oxygen support weaning and length of hospitalization. As a third endpoint, we correlated these findings with coagulation (VWF:Ag, FVIII, protein C, FVIII/protein C, D-dimer), inflammatory (IL-6, sC5b9, C5a) and endothelial perturbation biomarkers (soluble endothelial selectin (sE-selectin), thrombomodulin, soluble endothelial protein C receptor (sEPCR), vascular endothelial growth factor (VEGF), intercellular adhesion molecule-1 (ICAM-1), vascular cell adhesion molecule-1 (VCAM-1). Finally, we sought to identify whether some of these variables were associated with worse prognosis in our population of low-intensity-care COVID-19 patients.

With the limitations of the reduced sample size compared with the initial established sample size calculation, we found that none of our patients had signs of DVT at CUS and that the two LUS scores were comparable with each other but were not prognostic of mortality, length of stay or time to oxygen support weaning. Among the humoral biomarkers, we found that VCAM-1 had a prognostic role for mortality in a multivariate model.

## 2. Materials and Methods

### 2.1. Study Design

This was an observational, single-centre, prospective nonpharmacological, no-profit study conducted in COVID-19 patients admitted at the emergency department of Fondazione IRCCS Ca’ Granda, Ospedale Maggiore Policlinico, Milan, Italy, and hospitalized in the Internal Medicine—Hemostasis and Thrombosis Unit from 26 January 2021 to 26 May 2021. The “PRevalence and INCIdence of deeP vein thrombosis and Lung UltraSound alterations in COVID-19 Patients hospitalized in a low-intensity care unit” [PRINCIPLUS study] was approved by the Ethics Committee of our Hospital (Milano Area 2, n° 98_2021 of 20 January 2021 and subsequent amendment n° 590_2021 on 19 May 2021) and was performed according to the 2013 revision of the Declaration of Helsinki and the code of Good Clinical Practice. 

### 2.2. Patients

Consecutive adult patients with a diagnosis of symptomatic COVID-19 defined as the presence of suggestive systemic symptoms (fever, dyspnoea, cough, respiratory failure) and positive polymerase chain reaction (PCR) for SARS-CoV-2 nasopharyngeal swab were included. Informed consent was obtained from each patient. Patients were eligible within 72 h of admission to the emergency department. Patients with age < 18 years, admitted from departments other than the emergency department, with a reduced expectancy of life (<3 months) due to diseases other than COVID-19 and unable to express consent were excluded.

### 2.3. Variables and Outcomes

At admission, demographic data, clinical characteristics, clinical history, home therapy and routine laboratory parameters were systematically collected for each patient. Routine laboratory tests collected within 72 h of admission to the ward were performed at the central laboratory of IRCCS Policlinico. Multimorbidity was assessed via the Charlson comorbidity index [12]. The arterial partial pressure of oxygen (PaO_2_) and the fraction of inspired oxygen (FiO_2_) ratio (P/F ratio) were used to assess respiratory failure. Outcomes were in-hospital mortality, length of stay in days and time to oxygen support weaning. In particular, the O_2_ weaning followed our internal protocol. Briefly, the targets were: P/F ratio > 250 for >24 h, SpO_2_ > 95% and RR < 30 acts breaths per min. If the patient was wearing continuous positive airway pressure (CPAP) helmet, the positive end-expiratory pressure (PEEP) levels were reduced by 2 cm H_2_O until a weaning to high flow nasal cannula (HFNC) 60 L/min; then, the HFNC flow was reduced to 40 L/min until nasal cannula could be used. None of the patients underwent proning during their hospital stay in our ward.

### 2.4. Compressive Ultrasound (CUS) Protocol

All eligible patients underwent bedside bilateral two-point lower limb CUS, using a GE Logiq-E ultrasound machine with a 7.5-MHz 12 L linear probe. All compressions were performed using B-mode imaging with transverse views by applying compression along the deep venous system of each patient. The examination was classified as positive for DVT in the presence of signs of femoral and/or popliteal thrombosis [13].

### 2.5. Lung Ultrasound (LUS) Protocol 

Every examination was performed with the patient in a sitting or standing position, with the C1-5 convex probe, using either FAST or abdominal presets. The exams were performed using the same equipment used for CUS (GE Logiq-E). The depth was set at 10–15 cm. The focal point was set on the pleural line. Frequency was set at 3 MHz. Dynamic range was set at 75–85 dB and gain was 50–60%. Each examination was recorded and stored as pseudo-anonymized on an external hard disk. LUS was performed in a point-of-care standardized and reproducible way according to Soldati [14]. Namely, the protocol envisages scanning seven regions for each lung (3 posterior, 2 lateral and 2 anterior regions), for a total of 14 lung regions. For each LUS performed, a fixed sequence was followed as depicted in Supplementary Figure S1. The final score was calculated using the two scoring systems defined by Soldati [14] and Vetrugno [15]. In this fashion, we assigned every zone a point according to the parenchymal lesion (see Table 1). The total score is the sum of the scores for each lung that is given by the sum of the scores for each region.

LUS was performed for each enrolled patient by two experienced clinicians or by one expert operator and a young operator from the Internal Medicine ward. A clinician was considered an expert when having at least 2 years of daily LUS expertise. We considered that 25 LUS examinations supervised by experts would be enough for training physicians without expertise in LUS [16]. Physicians with less experience also attended a four-hour course on frontal lessons. The first operator performed the examination, and the second operator was present during the exam and evaluated the video. The two decided the scores independently and compared their results. Cases of disagreement were discussed to decide the final result. 

### 2.6. Humoral Biomarkers

Coagulation, inflammatory and endothelial biomarkers were tested at the Angelo Bianchi Bonomi Hemophilia and Thrombosis Center of our institution. For each patient EDTA, sodium citrate plasma and serum samples were collected within 72 h of admission. The samples were centrifuged at 2000× *g* for 15 min at room temperature, and the plasma aliquots were immediately frozen and stored at −80 °C until testing. 

Factor VIII activity was determined by performing a modified activated partial thromboplastin time test, using HemosIL Factor FVIII deficient plasma and SynthASil (Werfen, Barcelona, Spain). D-Dimer was assessed by means of D-Dimer HS 500 HemoSil, an automated latex immunoassay (Werfen). Von Willebrand factor (VWF) antigen was measured using an automated latex enhanced immunoassay (HemosIL Von Willebrand Factor Antigen, Werfen). Plasma levels of protein C were measured as chromogenic activity with the HemosIL Protein C kit (Werfen). All the above parameters were measured with the ACLTop 500 coagulation analyzer (Werfen). Tissue-type plasminogen activator (t-PA) antigen was measured in plasma using a commercially available ELISA (Zymutest t-PA antigen, Hyphen BioMed, Neuville sur Oise, France). The intra- and inter-assay CVs were <10%, and the lower detection limit was 0.5 ng/mL. Plasminogen activator inhibitor-1 (PAI-1) activity was detected using a commercial immunoassay (Zymutest PAI-1 activity; Hyphen BioMed) with intra- and inter-assay CVs of 3.5 and 5.6%.

IL-6 serum concentration was detected using the Human IL-6 Quantikine ELISA Kit (R&D Systems), which has intra- and inter-assay CVs respectively of 2.6% and 4.5%; the lower detection limit was 0.70 pg/mL. 

Complement system proteins C3 and C4 were measured using radial immunodiffusion (RID; NOR-Partigen, Siemens Healthcare Diagnostics, Munich, Germany). Blood samples were collected into EDTA tubes for the measurement of complement activation products. The plasma levels of soluble C5b-9 (SC5b-9) were measured using a solid-phase assay (MicroVue Complement SC5b-9 Plus EIA kit, Quidel Corporation, San Diego, CA, USA) whose intra- and inter-assay coefficients of variation (CVs) respectively were 6.8% and 13.1%; the lower detection limit was 3.7 ng/mL. Plasma C5a levels were measured using an immunoenzymatic method (MicroVue Complement C5a EIA, Quidel Corporation) with intra- and inter-assay CVs of <12%; the lower detection limit was 0.01 ng/mL.

sE-selectin was measured in plasma using a sandwich ELISA (Human sE-Selectin/CD62E Quantikine ELISA Kit, R&D Systems Minneapolis, MN, USA) whose intra- and inter-assay CVs respectively were 5.9% and 7.8%; the lower detection limit was 0.009 ng/mL. Soluble plasma thrombomodulin (sTM) levels were measured using a commercial sandwich ELISA (Human Thrombomodulin/BDCA-3 Quantikine ELISA Kit, R&D Systems), whose intra- and inter-assay CVs were 2.9% and 6.9%; the lower detection limit was 7.82 pg/mL. sEPCR plasma levels were measured using the Human EPCR DuoSet ELISA (R&D Systems), which has an inter-assay CV of 6.6% and a lower detection limit of 0.064 ng/mL. VEGF in serum was assessed using the Human VEGF Quantikine ELISA Kit (R&D Systems), whose intra- and inter-assay CVs respectively were 5.4% and 7.3%; the lower detection limit was 9.0 pg/mL. ICAM-1 was measured in serum using the Human ICAM-1/CD54 Allele-specific Quantikine ELISA Kit (R&D Systems), whose intra- and inter-assay CVs were, respectively, 4.6% and 5.5%; the lower detection limit was 0.096 ng/mL. VCAM-1 was assessed in serum by means of the Human VCAM-1/CD106 Quantikine ELISA Kit (R&D Systems). Intra- and inter-assay CVs were, respectively, 3.1% and 7.0%, while the lower detection limit was 0.6 ng/mL.

### 2.7. Statistical Analysis

Continuous variables were expressed as mean and standard deviation (SD) if distributed normally or as median and interquartile range (IQR) if distributed non-normally, while categorical variables were expressed as absolute numbers and percentages. The Spearman correlation coefficient was used to quantify the strength of the relationship between the Soldati and Vetrugno LUS scores.

To investigate factors associated with mortality, we performed a backward stepwise logistic regression analysis starting with a full model containing the following variables: age, sex, Charlson score, P/F ratio, RR, LUS scores, thrombomodulin, D-dimer, and VCAM-1. A nominal α level of 0.005 as cut-off for variable selection was considered. The results are reported in terms of odds ratios (ORs) and their relative confidence intervals (CIs). A linear regression analysis was used to determine the factors that were related to the time to oxygen support weaning and length of stay. Toward this aim, given that both outcomes were highly skewed, i.e., non-normal, we applied a logarithmic transformation to normalize the data to fulfil the assumptions of the linear regression model. Thus, regression coefficients should be interpreted as the effect of a unitary increase in a given covariate, i.e., demographic variable or endothelial or coagulation biomarker, on the natural logarithm of the time to oxygen support weaning and length of stay.

### 2.8. Sample Size Calculation

We calculated a sample size population and planned to enrol 100 patients with COVID-19 hospitalized in our low-intensity care ward, allowing us to detect a number of DVT events equal to 7% of the subjects, with precision, i.e., half-width of the confidence interval, equal to 5% and a confidence level of 95%, according to literature data [17,18]. 

### 2.9. Potential Bias

In consideration of the semi-quantitative nature of LUS, in order to avoid subjective interpretations in assigning scores to lung regions, we decided to perform each ultrasound study, both CUS and LUS, in the presence of at least two experienced physicians. In most cases, it was not the treating clinician of the actual patient who carried out the ultrasound study. However, the ultrasound result was not blinded.

## 3. Results 

### 3.1. Patients

**Figure 1:**
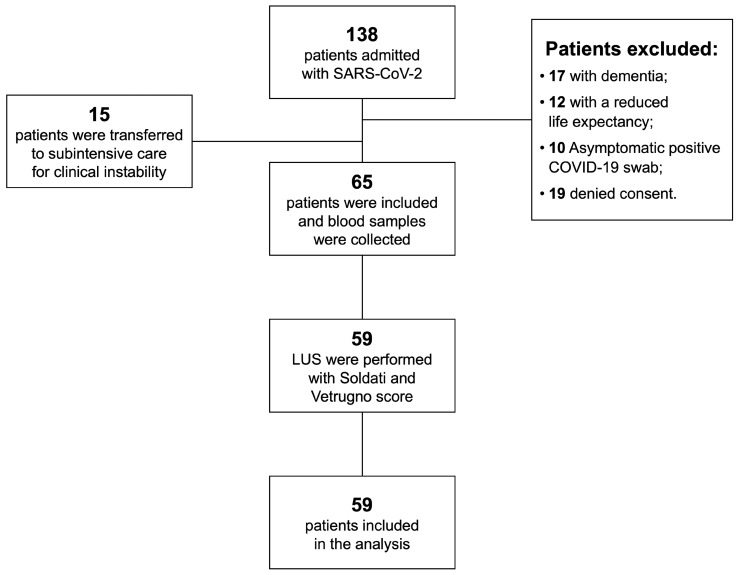
Flow diagram of the enrolment of the study population.

A total of 138 patients were admitted to the emergency department between 26 January 2021 and 26 May 2021 with SARS-CoV-2 infection, 73 of whom presented with at least 1 exclusion criterion; therefore, 65 were finally included in this study (Figure 1).

Patients’ characteristics and ongoing treatment on admission in survivors and nonsurvivors are reported in Table 2. The mean age (SD) was 69.7 (15.1) years, and 37 (57%) were men. The only consistently different variables in the two groups were age and the Charlson comorbidity index, which also includes age as a variable. The various comorbidities were not substantially different in the two groups. No significant difference was found in the chronic treatment at baseline between the two survival groups (Table 2). On admission, a proportion of patients was already taking corticosteroids (29.2%), anticoagulant therapy (6.2%) or heparin prophylaxis (21.5%). The mean time from onset of symptoms to admission was 7.5 days, while the mean time from swab positivity to admission was 4.4.

Laboratory findings at baseline are expressed in Table 3. Although the means of the laboratory parameters were different between survivors and non-survivors, only creatinine levels showed a statistically significant difference.

### 3.2. Outcome Data

In our cohort, 8 patients (12%) died during hospital stay, 27 (41.5%) needed oxygen administration without noninvasive ventilation (NIV) and 30 (46.2%) needed oxygen administration with NIV. In the NIV group, 11 were treated with HFNC (36.7%), and 19 needed CPAP ventilation with a helmet (63.3%). All eight non-survivors underwent NIV except for one patient who had advanced lung cancer (3.3% of NIV patients); two patients received NIV with HFNC (6.7% of total NIV patients), and five received CPAP (16.7% of total NIV patients). The mean (SD) days on oxygen therapy was 12.3 (10.8 [1–63]). The mean (SD) days alive in the non-survivor group was 20.3 (8.5). The mean (SD; [min–max]) length of hospital stay was 15.8 (13.4, [0–77]).

### 3.3. CUS Results

Of the 65 patients recruited in our study, CUS and LUS were performed within 72 h of admission in 59 patients. Of these, 18 (30%) were on anticoagulant treatment: 4 patients were on chronic anticoagulation, and 14 patients were on heparin prophylaxis prescribed for COVID-19 by the general practitioner. None of the femoral-popliteal CUS performed showed the presence of DVT.

### 3.4. LUS Results

LUS performed at hospitalization showed that most patients had an inflammatory pattern in at least 2 lung regions, with a LUS score (both assessed with Soldati and Vetrugno scores) of >6 points in 96.6% of cases. In particular, the most prevalent pattern in our cohort was B2 with both scores. Moreover, a LUS score >15 points, a proposed cut-off for LUS severity [19,20], was found in 71.2% and 55.9% of patients assessed with Soldati and Vetrugno score, respectively (Table 4). The two LUS scores were strongly correlated with each other (rho = 0.95).

We found no statistically significant difference between survivors and non-survivors regarding both LUS scores. We then analysed differences in LUS scores in patients who needed oxygen therapy or NIV vs. those who did not and found a significant difference for both scores (Soldati score *p* = 0.017; Vetrugno score *p* = 0.023, Table 4). 

### 3.5. Humoral Biomarkers

The results for the biomarkers tested in the blood samples of all 65 patients are reported in Table 5. D-dimer mean (SD) levels were higher in non-survivors 1165.6 ng/mL (532.5 ng/mL) than in survivors 846.6 ng/mL (564.9 ng/mL), but the results were not statistically significant (*p* = 0.144). Mean (SD) thrombomodulin levels in non-survivors were 7283.9 pg/mL (3961.6 pg/mL) vs. 4800.7 pg/mL (1771.0 pg/mL) in survivors, and this difference was statistically significant (*p* = 0.004; Table 5). Mean (SD) VCAM-1 levels in nonsurvivors were higher than in survivors (2299 ng/mL (730.5 ng/mL) vs. 1451 ng/mL (456.2 ng/mL), *p* < 0.001). IL-6, FVIII, VWF, sEPCR, sC5b9, tPA, PAI-1 activity, C4 and ICAM-1 levels were higher than normal ranges but were not substantially different between survivors and non-survivors (Table 5). We further analysed differences in laboratory parameters between those who never needed oxygen and those who needed oxygen therapy or noninvasive ventilation (NIV). Among the endothelial biomarkers, only VEGF showed statistical significance, with mean (SD) levels lower at baseline in patients who did not need oxygen during hospitalization [281.9 pg/mL (124.1 pg/mL)] than in patients who needed oxygen therapy [479.5 pg/mL (330. pg/mL)] (*p* = 0.047).

### 3.6. Univariate and Multivariate Regression Analysis

Univariate linear and logistic regression were performed with length of stay, time to oxygen support weaning and mortality as outcomes (Table 6). The first two outcomes were not normally distributed (skewed) and therefore were normalised by logarithmic transformation. Age, creatinine, P/F ratio, respiratory rate (RR) as breaths per minute and D-dimer were statistically significant for length of stay and time to oxygen support weaning. VCAM-1 was significant for time to oxygen support weaning and death. Charlson score and thrombomodulin levels were significant for all the measures of outcome (see Table 6).

In a multivariate linear and logistic analysis, we chose to include age, sex, Charlson score, RR, P/F ratio and VCAM-1 as significant variables and to include these together with LUS score (Table 7). The Soldati and Vetrugno scores are strongly correlated (rho = 0.95) and therefore cannot be considered in the same model because this would lead to a multicollinearity problem. In a model with the LUS Vetrugno score, VCAM-1 level increase was associated with death. The model with the Soldati score yielded comparable results.

## 4. Discussion

In our cohort of 65 patients with COVID-19 admitted in a non-ICU ward, we conducted a cross-sectional study to investigate ultrasonographic features of lungs and vessels (CUS and LUS), coagulation, inflammation and endothelial perturbation biomarkers on hospital admission. 

We found no DVT in our cohort of patients, despite the presence of highly abnormal inflammatory and coagulation markers. COVID-19 is characterized by a state of hypercoagulability that is reported to be associated with DVT and PE. However, none of the CUS performed at the time of hospitalization in our cohort was positive for DVT. We contend that the main reason for these findings was that during the second wave of the pandemic, heparin and steroids were prescribed by general practitioners in the metropolitan area of our hospital. In addition, no patient was bedridden or had recent surgery. Recent studies have highlighted that COVID-19 patients have significantly increased thrombin generation that can be reduced to that of healthy controls if thromboprophylaxis with heparin is administered [21]. Moreover, heparin treatment improved survival in severe COVID-19 patients with high D-dimer values [22]. Despite the absence of DVT, our population showed elevated D-dimer levels in the whole cohort with higher levels in non-survivors. So far, it has been shown that the incidence of PE is increased in COVID-19 patients, particularly in those with high D-dimer, without a marked increase in the incidence of DVT [23]. In addition, pathological findings of in situ microvascular thrombosis with microangiopathy and the occlusion of alveolar capillaries [24] showed a lesser extent also in the heart [25] and kidneys [26]. These findings are often found in COVID-19 autopsies, and microvascular thrombosis was found nine times more frequently than in the classic ARDS from H1N1 influenza A virus [27]. Radiological findings report a higher frequency of distal thrombotic lesions in the lung vasculature, suggesting that they may reflect in situ pulmonary thrombosis instead of the typical manifestations of a thromboembolic disease [24]. Another relevant finding is that of platelet-rich thrombi in the pulmonary, hepatic, renal and cardiac microvasculature in COVID-19 autopsies [28], suggesting the local activation of thrombosis rather than thromboembolism.

For the LUS examinations, our findings indicate that the majority of COVID-19 patients admitted to our unit presented an ultrasonographic pattern of lung injury typical of interstitial pneumoniae, even without the need for oxygen therapy. LUS findings at baseline could not discriminate the outcomes of patients in terms of survival, but a significant difference was found between patients needing and not oxygen therapy with both scores. However, the operator performing LUS was sometimes the same prescribing oxygen therapy; therefore, we cannot exclude a selection bias for NIV treatment. 

The mean time from symptom onset in our population was 7 days, i.e., halfway between the replicatory and inflammatory phases of disease. As LUS was performed within 72 h of hospital admission, perhaps it was too early for lung injury to develop. Soldati et al. give a higher score for the interstitial pattern (pattern B2), whereas Vetrugno et al. give a higher score for consolidations (pattern C) [14,15]. However, COVID-19 causes interstitial pneumonia, and accordingly, the prevalent type of lung injury for both LUS scores was B2 pattern. Different studies have proposed a threshold for LUS score in association with worse outcome [19]. In our cohort, mean and median LUS score were >15 in both survivors and non-survivors, as assessed by both scores. We were not able to define a significant cut-off because of our relatively small sample size, which underpowered the study.

Regarding the inflammatory, coagulation and endothelial perturbation biomarkers, most clinical studies have examined elevation in endothelial biomarkers mainly in an ICU setting [29,30]. A few articles provided some biochemical evidence of endotheliopathy in non-ICU patients, demonstrating elevation of VWF:Ag, P-selectin and thrombomodulin and showing that higher levels of these markers were associated with worse outcomes [4]. In the present cohort of COVID-19 patients, thrombomodulin, IL-6, FVIII, VWF:Ag, sEPCR, sC5b9, tPA, PAI-1 activity, C4, ICAM-1 and VCAM-1 plasma levels were higher than normal ranges, in line with previous studies that included patients with a severe disease [4,5,29].

In particular, soluble thrombomodulin and VCAM-1 plasma levels were significantly different between the two survival groups. Thrombomodulin is an endothelial transmembrane glycoprotein that binds thrombin, increasing C protein activation as an anticoagulant factor. Soluble thrombomodulin is intended as an endothelial damage marker because its increased value in serum is related to endothelial cell shedding or disruption. Similar to our results, several studies found that elevated levels of thrombomodulin were associated with longer hospital stay [4,31]. VCAM-1 is another biomarker of endothelial perturbation; in particular, it is a glycoprotein expressed at the endothelial cell surface level whose expression is increased by pro-inflammatory cytokines, oxidative stress, high glucose concentration, toll-like receptor agonists and shear stress [32]. High plasma levels have been observed in COVID-19 patients and are associated with disease progression and mortality [33,34,35,36]. 

In the present study, on univariate linear regression, thrombomodulin plasma levels on admission were associated with length of stay, time to oxygen support weaning and death; VCAM-1 levels were significantly associated with time to oxygen support weaning and death (Table 6).

The fact that inflammatory and coagulation activation biomarkers such as C reactive protein and D-dimer were not significantly different in survivors and non-survivors, could be due to the fact that patients were already being treated by general practitioners with steroids and heparin and therefore presented a less pronounced inflammatory component compared with patients from the first pandemic wave.

As for the other endothelial damage markers, we hypothesize that the relatively small sample size reduced the statistical power of our study, not allowing us to find a statistically significant difference but only a descriptive trend between the two survival groups and between the patients with or without the need for oxygen. 

### Limitations and Bias

Our study should be interpreted considering several limitations. First, it was a cross-sectional study with data collected during the pandemic. 

Second, although we pre-established a sample size of 100 patients, this target was not reached due to the limited number of patients transferred from the ED (based on the inclusion criteria) and due to the inability of some of them to sign informed consent as well as refusal by some others. Patients transferred from other departments were also not included in the study. Third, it is not possible to exclude the presence of treatment bias due to the fact that the physician prescribing the oxygen therapy was not blinded to the LUS results. Fourth, the second wave of the pandemic was characterized by a more solid and established treatment approach since patients hospitalized were often ongoing thromboprophylaxis or steroid treatment already prescribed by general practitioner and therefore reducing the differences between patients in terms of inflammation and thrombotic risk.

Finally, we enrolled only a population admitted at a low-intensity-care clinical ward, and therefore we have no ICU population as a possible comparison group.

## 5. Conclusions

In a cohort of mild COVID-19 patients, we found no DVT events despite the presence of highly abnormal inflammatory, endothelial perturbation and coagulation biomarkers, thus supporting the hypothesis that COVID-19 pathogenesis is due to relevant endotheliopathy. We observed that LUS is a useful tool for describing and assessing the typical pattern of interstitial pneumonia in COVID-19. The B2 pattern is associated with more severe disease. LUS abnormalities can be found in early stage of the disease, but the total score at admission was not useful as an early prognostic instrument. Finally, VCAM-1 emerged as a promising prognostic biomarker for mortality in COVID-19. Given the high availability and simple use of the existing ELISA kits, the measurement of VCAM-1 can be considered in the routine laboratory panel of COVID-19 patients on admission.

Our study was performed in a complex historical moment and expresses the value of collaboration for correlating clinical, ultrasound, laboratory and nonroutine parameters, markers of endothelial perturbation. The study was conducted in an effort to provide insights into the pathogenesis, clinical features and prognostic factors of COVID-19 patients with the support of a multidisciplinary team.

## Figures and Tables

**Table 1:** Scoring systems for lung ultrasound by Soldati et al. and Vetrugno et al. [14,15].

	Soldati Scoring System	Vetrugno Scoring System
Score 0	Normal (A-lines)	Normal (A-lines)
Score 1	Jagged pleural line with few B-lines departing (B1 pattern)	B1 pattern with at least 3 B lines
Score 2	Presence of lung consolidations	B2 pattern with coalescent B lines
Score 3	Presence of dense and fused B lines in the shape of B2 pattern “white lung”	Presence of supra-centimetre consolidations

**Table 2:** Characteristics of patients on admission.

	All Patients (N = 65)	Survivors (N = 57)	Non-Survivors (N = 8)
Demographic characteristics			
Age (years), mean (SD)	69.7 (15.1)	68.2 (15.5)	79.8 (5.6) *
Age, range	32–92	32–92	72–90
Male, n (%)	37 (56.9)	34 (59.6)	3 (37.5)
Charlson Comorbidity Index ^#^, mean (SD)	4.1 (2.2)	3.8 (2.0)	6.8 (1.7) **
Drugs			
Angiotensin converting enzyme inhibitor/receptor blocker, n (%)	23 (35.4)	20 (35.0)	3 (37.5)
Anticoagulant (VKA, DOAC), n (%)	4 (6.2)	4 (7.0)	0 (0.0)
Heparin, n (%)	14 (21.5)	12 (21.0)	2 (25.0)
Steroids, n (%)	19 (29.2)	17 (29.8)	2 (25.0)

DOAC Direct oral anticoagulant; SD standard deviation; VKA Vitamin K antagonist. ^#^ Charlson Comorbidity Index is a composite index including age, myocardial infarction, chronic heart failure, peripheral vascular disease, cerebrovascular accident or TIA, dementia, chronic obstructive pulmonary disease, connective tissue disease, peptic ulcer disease, liver disease, diabetes mellitus, hemiplegia, moderate to severe chronic kidney disease, solid tumour, leukaemia, lymphoma and acquired immune deficiency syndrome. * *p* < 0.005, ** *p* < 0.001.

**Table 3:** Laboratory findings at baseline.

	All Patients (N = 65)	Survivors (N = 57)	Non-Survivors (N = 8)	*p* Value
Arterial blood gas analysis examination				
SatO_2_ (%), mean (SD)	97.0 (2.1)	97.3 (1.9)	96.4 (2.8)	0.304
pH, mean (SD)	7.5 (0.04)	7.5 (0.04)	7.5 (0.05)	0.867
PaO_2_, mmHg, mean (SD)	81.6 (14.7)	81.9 (15.1)	79.4 (12.5)	0.650
PaCO_2_, mmHg, mean (SD)	33.7 (4.3)	34.0 (4.3)	31.4 (4.2)	0.104
PaO_2_/FiO_2_, mmHg, mean (SD)	308 (76.3)	310 (73.9)	389 (94.7)	0.464
Complete blood count				
White blood cells, ×10^6^/L, mean (SD)	6769 (3245.8)	6612 (2913)	7887 (5175.7)	0.302
Lymphocytes, ×10^9^/L, mean (SD)	1212.3 (1432)	1026.3 (551)	2515.0 (3723.8)	0.005
Haemoglobin, g/dL, mean (SD)	12.6 (2.2)	12.7 (2.2)	12.4 (1.9)	0.772
Platelets, ×10^9^/L, mean (SD)	194.1 (91.4)	192.4 (90.6)	206.8 (102.7)	0.680
Biochemistry				
C-reactive Protein, mg/dL, mean (SD)	5.8 (5.2)	5.8 (5.1)	5.9 (5.9)	0.948
LDH, U/L, mean (SD)	271.6 (83.2)	267.2 (82)	305.3 (89.2)	0.257
ALT, U/L, mean (SD)	31.9 (28.1)	32.5 (29.9)	27.8 (8.9)	0.657
Creatinine, mg/dL, mean (SD)	1.1 (0.8)	0.98 (0.35)	1.99 (2)	0.001
PT ratio, mean (SD)	1.1 (0.2)	1.1 (0.2)	1.0 (0.07)	0.290
PTT ratio, mean (SD)	0.9 (0.2)	0.9 (0.3)	0.95 (0.07)	0.855
Fibrinogen, mg/dL, mean (SD)	512.6 (130.5)	516 (134.8)	492.5 (109.1)	0.688
Ferritin ng/mL, mean (SD)	706.9 (648.1)	735.4 (683.9)	507.4 (233)	0.356

PaO_2_: arterial partial pressure of oxygen, PaCO_2_ partial pressure of carbon dioxide, FiO_2_: fraction of inspired oxygen, SD standard deviation.

**Table 4:** Lung ultrasound (LUS) findings at baseline in survivors vs. non-survivors and in patients treated vs. patients not treated with oxygen therapy.

	All Patients (N = 65)	Survivors (N = 57)	Non-Survivors (N = 8)	*p* Value	No Oxygen Therapy (N = 8)	Oxygen Therapy and NIV (N = 57)	*p* Value
SOLDATI total LUS score mean (SD)	20.2 (8.4)	19.8 (8.0)	23.1 (11.6)	0.335	13.6 (6.9)	21.3 (8.2)	0.017
VETRUGNO total LUS score mean (SD)	16.4 (6.4)	16.2 (6.1)	17.9 (9.2)	0.461	12.0 (4.3)	17.0 (6.5)	0.023

**Table 5:** Humoral biomarker levels in survivors vs. non-survivors.

	All Patients (N = 65)	Survivors (N = 57)	Non-Survivors (N = 8)	*p* Value	Reference Values
sE-selectin (ng/mL), mean (SD)	25.0 (15.6)	24.9 (15.9)	25.5 (15.2)	0.931	13.0–51.03
**Thrombomodulin (pg/mL), mean (SD)**	**5143.2 (2317.3)**	**4800.7 (1771.0)**	**7283.9 (3961.6)**	**0.004**	**2353–4541**
IL-6 (pg/mL), mean (SD)	44.8 (102.4)	40.3 (102.8)	73.1 (101.9)	0.404	<10
FVIII (%), mean (SD)	160.6 (57.4)	164.8 (59.7)	136.1 (35.7)	0.194	54–133
PC (%), mean (SD)	103.8 (25.7)	104.5 (25.7)	99.9 (26.7)	0.640	84–145
VWF:Ag (%), mean (SD)	251.8 (90.9)	245.3 (83.4)	288.9 (126.6)	0.215	70–194
D-dimer standardized (ng/mL), mean (SD)	893.9 (567.0)	846.6 (564.9)	1165.6 (532.5)	0.144	<500
FVIII/Protein C, mean (SD)	1.578 (0.504)	1.610 (0.527)	1.394 (0.313)	0.268	1
sEPCR (ng/mL), mean (SD)	80.13 (49.0)	76.5 (47.3)	100.9 (56)	0.197	12–64
VEGF (pg/mL), mean (SD)	455.6 (318.5)	435.9 (316.9)	579.1 (320.7)	0.241	62–707
sC5b9 (ng/mL), mean (SD)	472.5 (389.5)	483.2 (410.5)	405.1 (223.3)	0.603	139–463
C5a (ng/mL), mean (SD)	16.2 (5.9)	16.1 (5.7)	16.6 (7.5)	0.819	0.37–74
tPA (ng/mL), mean (SD)	10.9 (6.2)	10.6 (5.8)	12.9 (8.3)	0.340	<10
PAI-1 activity (ng/mL), mean (SD)	10.2 (28.8)	11.5 (30.8)	2.5 (4.3)	0.420	<5
C3c (%), mean (SD)	122 (20.2)	123 (20.3)	116 (1990)	0.403	70–130
C4 (%), mean (SD)	180 (52.7)	180 (51.7)	179 (62.6)	0.961	60–140
ICAM-1 (ng/mL), mean (SD)	324 (110.6)	325 (116.7)	318 (65.9)	0.864	99–320
**VCAM-1 (ng/mL), mean (SD)**	**1568 (575.8)**	**1451 (456.2)**	**2299 (730.5)**	**<0.001**	**349–991**

In bold are the humoral biomarkers with a statistically significant difference (*p* < 0.05). PC protein C, IL-6 interleukin-6, sEPCR soluble endothelial protein C receptor, FVIII coagulation factor VIII, VEGF vascular endothelial growth factor, VCAM-1vascular cell adhesion molecule-1, ICAM-1 intercellular adhesion molecule-1, PAI-1 Plasminogen activator inhibitor—1, tPA tissue plasminogen activator, VWF:Ag Von Willebrand factor antigen.

**Table 6:** Univariate analysis of prognostic factors and different outcomes.

	Length of Stay °°	Time to Oxygen Support Weaning °°	Death °
Age	0.02 (0.01, 0.03) **	0.03 (0.01, 0.04) **	1.07 (1.01, 1.16) *
Charlson score	0.13 (0.06, 0.20) **	0.13 (0.03, 0.22) *	1.91 (1.32, 3.08) *
Creatinine	0.02 (0.00, 0.04) *	0.03 (0.00, 0.05) *	1.15 (1.03, 1.35) *
CPR	0.45 (0.13, 0.78) *	0.33 (−0.09, 0.76)	1.05 (0.18, 3.67)
LDH	0.02 (0.00, 0.05) *	0.03 (0.00, 0.05)	1.05 (0.96, 1.15)
P/F ratio	−0.29 (−0.52, −0.01) *	−0.47 (−0.79, −0.15) *	0.69 (0.24, 1.84)
RR	0.21 (0.05, 0.36) *	0.24 (0.03, 0.45) *	1.33 (0.66, 2.64)
Thrombomodulin	0.09 (0.01, 0.16) *	0.13 (0.03, 0.22) *	1.44 (1.08, 2.08) *
SC5b9	0.06 (0.01, 0.10) *	0.02 (−0.04, 0.09)	0.93 (0.66, 1.13)
D-dimer	0.03 (0.00, 0.06) *	0.05 (0.01, 0.10) *	1.09 (0.96, 1.23)
VCAM-1	0.02 (−0.01, 0.05)	0.05 (0.01, 0.09) *	1.31 (1.12, 1.63) *
VWF:Ag	−0.01 (−0.21, 0.19)	0.06 (−0.24, 0.36)	1.53 (0.72, 2.98)
ICAM-1	0.12 (−0.04, 0.27)	0.22 (−0.01, 0.46)	0.94 (0.42, 1.79)
PAI-1	0.00 (−0.01, 0.00)	0.00 (−0.01, 0.01)	0.96 (0.80, 1.01)
FVIII	0.01 (−0.02, 0.04)	0.01 (−0.03, 0.06)	0.89 (0.73, 1.04)
C5a	0.02 (−0.01, 0.05)	0.01 (−0.04, 0.05)	1.02 (0.90, 1.17)
tPA	0.03 (0.00, 0.05)	0.03 (0.00, 0.07)	1.06 (0.94, 1.19)

CPR C reactive protein, FVIII coagulation factor VIII, VCAM-1vascular cell adhesion molecule-1, ICAM-1 intercellular adhesion molecule-1, PAI-1 Plasminogen activator inhibitor—1, P/F ratio arterial oxygen partial pressure (PaO_2_) to fractional inspired oxygen (FiO_2_) ratio, RR respiratory rate, tPA tissue plasminogen activator, VWF:Ag Von Willebrand Factor antigen. °° β estimates with 95% CI are reported (linear regression). ° odds ratios with 95% CI are reported (logistic regression). ** *p* < 0.001. * *p* < 0.05.

**Table 7:** Multivariate analysis of prognostic factors.

	Length of Stay °°	Time to Oxygen Support Weaning °°	Death °
Vetrugno LUS score	0.00 (−0.02, 0.03)	0.00 (−0.03, 0.04)	1.19 (0.95, 1.66)
P/F ratio	Not included	−0.47 (−0.85, −0.09) *	Not included
RR	0.32 (0.14, 0.51) **	0.23 (0.01, 0.45) *	Not included
VCAM-1	Not included	Not included	1.31 (1.06, 1.81) *

P/F ratio arterial oxygen partial pressure (PaO_2_) to fractional inspired oxygen (FiO_2_) ratio; RR respiratory rate; VCAM-1 1vascular cell adhesion molecule-1. Data reported are adjusted for age, sex and Charlson score. °° β estimates with 95% CI are reported (linear regression). ° odds ratios with 95% CI are reported (logistic regression). ** *p* < 0.001. * *p* < 0.05.

## Data Availability

The data presented in this study are available on request from the corresponding author. The data are not publicly available for privacy reasons.

## Supplementary Materials

PRINCIPLUS study group: Niccolò Bitto, Enrico Rino Bregani, Marigrazia Clerici, Michele De Caro, Elena Grovetti, Samantha Griffini, Erica Pagliaro, Gabriele Ghigliazza, Sofia Restelli, Erica Scalambrino. The following supporting information can be downloaded at: https://www.mdpi.com/article/10.3390/jcm11185425/s1, Figure S1: Lung ultrasound scanning sequence.
