# Supplementary figures and images for: Lung Ultrasound Findings and Endothelial Perturbation in a COVID-19 Low-Intensity Care Unit
Source: J Clin Med. 2022 Sep 15;11(18):5425. doi: 10.3390/jcm11185425 (PMC9504266; doi:10.3390/jcm11185425)

**Figure S1.** Lung ultrasound scanning sequence.

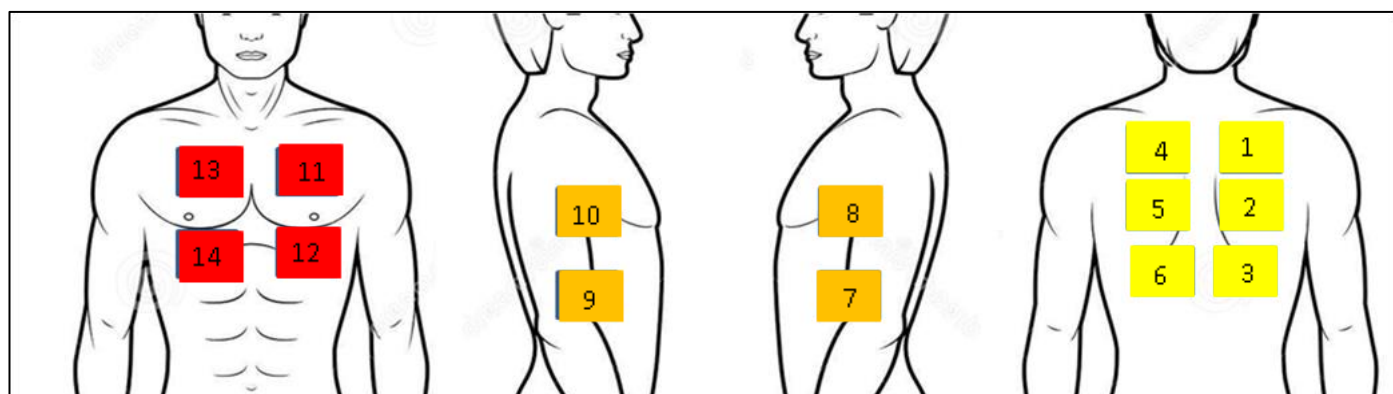

Supplement: Supplementary file 1 [file jcm-11-05425-s001.zip › jcm-1865137-supplementary.pdf]
